# Wearable Sensor Systems for Infants

**DOI:** 10.3390/s150203721

**Published:** 2015-02-05

**Authors:** Zhihua Zhu, Tao Liu, Guangyi Li, Tong Li, Yoshio Inoue

**Affiliations:** 1 The State Key Laboratory of Fluid Power Transmission and Control, Department of Mechanical Engeering, Zhejiang University, Hangzhou 310027, China; E-Mails: zhihuaZhu@zju.edu.cn (Z.Z.); li_guangyi@zju.edu.cn (G.L.); zjulitong@zju.edu.cn (T.L.); 2 School of Systems Engineering, Kochi University of Technology, Kochi 780-8520, Japan; E-Mail: inoue.yoshio@kochi-tech.ac.jp

**Keywords:** wearable sensor systems for infants, constant health monitoring, wearable technique, wearable sensor network, power supply, integrating and wireless sensor technologies

## Abstract

Continuous health status monitoring of infants is achieved with the development and fusion of wearable sensing technologies, wireless communication techniques and a low energy-consumption microprocessor with high performance data processing algorithms. As a clinical tool applied in the constant monitoring of physiological parameters of infants, wearable sensor systems for infants are able to transmit the information obtained inside an infant's body to clinicians or parents. Moreover, such systems with integrated sensors can perceive external threats such as falling or drowning and warn parents immediately. Firstly, the paper reviews some available wearable sensor systems for infants; secondly, we introduce the different modules of the framework in the sensor systems; lastly, the methods and techniques applied in the wearable sensor systems are summarized and discussed. The latest research and achievements have been highlighted in this paper and the meaningful applications in healthcare and behavior analysis are also presented. Moreover, we give a lucid perspective of the development of wearable sensor systems for infants in the future.

## 1. Introduction

In recent years, a wide range of wearable devices and sensors including accelerometers and gyroscopes, smart fabrics and actuators, wireless communication networks and power supplies, and data capture technology for processing and decision support [1], have been developed for clinical research and health monitoring. Various kinds of wearable sensors have emerged for different purposes with the development of sensing technologies. For instance, wearable devices, instrumented with variable resistance bend sensors, enable applications for human posture recognition and motion capture by recovering human joint bend angles [2]. Since pH shifts at the wound site can provide useful information regarding wound evolution, Guinovart *et al.*, introduced a new device which was based on the judicious incorporation of a screen-printed pH potentiometric sensor into bandages to monitor the pH of wounds and track the pH fluctuations with no apparent carry-over effect [3]. Another highly-sensitive and ultra-thin silicon stress sensor chip was developed by reconfiguration of individual sensor chips for thinning and lamination, which was used for the measurement of human pulses on wrist and tooth orthodontic forces of invisible aligners [4]. A wearable wrap-around sensor composed of multiple electrodes (antennas, excited at a single low frequency), was proposed for continuous measurement of the permittivity of biological tissues deep into the torso (specifically the lungs and heart) [5]. In our previous studies on human gait analysis, we also developed some wireless wearable sensor systems, such as the mobile force plate system, to implement quantitative human kinematic and kinetic analysis, which may be applied in rehabilitation, clinical diagnosis and healthcare monitoring in the future [6–8].

Health monitoring is an essential application of wearable sensor systems, especially for infants. Recently, together with advances in sensor techniques, wireless communication and power supply technologies, wearable sensor systems have enabled the creation of a new generation of constant health monitoring for infants. For example, a developed sensory baby vest including fully-integrated sensors for measuring electrocardiography (ECG), respiration, temperature and humidity (to detect excessive sweating), which allow the early detection of potential life-threatening events [9]. Conductive textile wires are used to make the sensors integration compatible in a prototype belt which is built of soft bamboo fabrics with a negative temperature coefficient (NTC) sensor to demonstrate temperature monitoring instead of hard wires in order to improve the comfort of the infant [10]. Sibrecht *et al.*, developed a neonatal smart expandable jacket that enables ECG measurement by textile electrodes and carried out several experiments to demonstrate the prototype [11]. Another example is a wearable multi-parameter monitor named BBA bootee, developed to achieve the monitoring of infants at risk of apparent life threatening events (ALTE). The sensors, electronics and the power supply are integrated into the bootee, which provides reliable pulse oximetry measurements as well as useful information about the infant's movement and position [12]. Through detecting the variation in the exhaled CO_2_ concentration using CO_2_ sensors placed in the crib around an infant, clinicians are able to detect if there is anything unusual with the infant's respiration [13]. In Yamada and Watanabe's study, they tried to construct a prototype of a small pressure-sensor-driven round bar grip measurement device called DataGrip to measure the infant's grip strength. With this grip force measuring system, they drew the conclusion that the infant's grip strength increased in correspondence with physical development [14]. Dedicated design and non-invasive sensor integration are necessary for the wearable sensor systems to achieve safe and reliable health monitoring.

Since these tiny, vulnerable infants can hardly articulate pain and uncomfortableness in Neonatal Intensive Care Units (NICU) or at home, continuous monitoring of their vital signs and physiological parameters is crucial for clinicians and parents to know their exact health conditions. Preterm infants or critically ill infants admitted into the NICU need sustained monitoring in case of various dangerous conditions, which may include apnea, hypoglycemia, sepsis or sepsis-like infection, seizure, arterial hypotonia, bradycardia, hypoxia, hypothermia, acidosis [15], and even problems of Sudden Infant Death Syndrome (SIDS). In addition, the apparent life-threatening events of 1- to 4-year-old infants, such as drowning in the bathrooms or head injuries occurring from falls or shaking, motivate clinicians and parents to search for an inexpensive and non-invasive way to effectively monitor the health status of infants and send alarm information whenever the infant's health condition is bad or whether the infant comes into contact with any other threatens from outside word.

The traditional method for monitoring infant health is usually carried out under the direct supervision of clinicians and parents. This method requires dedicated manpower and sometimes it is difficult for clinicians and parents to identify the infants' potential physiological condition. The initial purpose of the application of wearable sensor systems in infants health monitoring is to provide them everyday-centered care to ensure that the appropriate care or therapy can be given to the onset of complications, and reduce the cost of hospital-based clinical interventions and the burdens of parents at home. Health monitoring system is therefore a tool that provides early indication of changing patient status, and allows for early intervention, but is also a means by which the effect of interventions and therapies may be recorded, evaluated and controlled [15]. Ideally, these systems should be capable of carefully, conveniently and robustly monitoring infants in the NICU or at home while infants perform their daily activities (such as eating, sleeping, and natural communication with parents) without interfering significantly with their comfort. However, the traditional sensors and medical instruments cannot be used for wearable physiological monitoring applications, as they are difficult to wear for long periods of time, and they also cause discomfort to the wearer. Because of the limitations of technologies in sensors, wireless networks, and energy supply and so on, conventional wearable sensor systems are generally not adequate for the robust, long-term and comfortable monitoring of infants in real life conditions [16]. However, with the development of wearable sensor systems, including miniature biosensing devices, smart textiles, microelectronics and wireless communication, constant and precise monitoring of physiological parameters for infants is becoming more available and convenient. In addition, with more and more sufficient consideration of infant needs and clinical requirements, the application of integrated sensing and consumer electronics technology is improving the reliability and comfort of monitoring systems, which ensure the quality of life and long-term health prospects of the infants. Wearable electronics and intelligent textiles effectively avoid the disturbance to infants caused by conventional sensor techniques which may include skin irritation, hampering due to wires [17], interruption of sleep, and lack of communication with parents [18].

The primary purpose of the current paper is to review the current status of infant monitoring technology based on wearable sensor systems. In Section 2, we introduce the basic monitored biomedical parameters and the fundamental structure of infant monitoring systems, which are the basis of the wearable sensor system. The current state-of-the-art wearable sensor systems for infants and the research methods and primary results of their development are reviewed in Section 3. Section 4 concentrates on providing a comprehensive outlook for the various applications of wearable sensor systems for infants. The summaries and the future prospects are detailed in Section 5.

## 2. Materials of Wearable Sensor Systems for Infants

### 2.1. Monitored Biomedical Parameters

The wearable sensors measure and monitor the physiological vital signs and parameters of infants through typical sensing principles and transducers during neonatal intensive care in the NICU or at home. Such variations of parameters represent the potential changes inside the body, and will manifest through external physical characteristics changes. The human chemical parameters and physiological vital signs most often monitored during neonatal intensive care and the relative sensing principles and transducers are shown in Table 1 [15].

Table 1 shows the most commonly monitored vital signs and parameters for infants. The relative sensing principles and transducers which may apply to the wearable sensor system are also provided. In the NICU, the critical physiological parameters or vital signs for the infants are ECG, body temperature and respiration. The clinicians often make a diagnosis and take the appropriate measures according to the monitoring results when the infants are in a critical condition. Effective monitoring protects these pre-term infants from various kinds of threats, such as apnea, hypoglycemia, seizure, bradycardia, hypoxia and hypothermia. Apart from these monitored parameters, some researchers have tried to find other important physical parameters to achieve health monitoring. Since human body odor and foot odor usually consist of various volatile compounds that can indicate normal and abnormal biomedical activities in the body, some researchers have tried to evaluate human health status and give early warning signs when abnormal biomedical activities take place in the body through detection of a patient's body odor by utilizing a wearable electronic nose (consisting of chemical gas sensors) embedded in clothing [21,22]. In addition, wearable sensor systems can be used for some special cases in healthcare and patient monitoring. For example, the prototype of attaching a triaxial accelerometer sensor to an infant's clothing to detect whether the infant is sleeping on their back, side or stomach [23]. Another monitoring system measures the wave motion of water in a bathtub to prevent near-drowning injury to infants at home [24].

### 2.2. Framework of Wearable Sensor Systems for Infants

A wearable sensor system may encompass a wide variety of components: sensors, wearable materials, smart textiles, actuators, power supplies, wireless communication modules and links, control and processing units, an interface for the user, software, and advanced algorithms for data extraction and decision-making. Generally, wearable sensor systems for health monitoring need to achieve specific functions under strict medical criteria and significant hardware resource limitations. More specifically, a qualified wearable sensor system design needs to take several wearability criteria into account, for example, the outward appearance should meet aesthetic requirements, the weight and the size of the system need to be small, and it should not hinder the user's movements and actions, especially in the case of infants. In addition, radiation or other types of health hazards are not allowed [25].

To achieve reliable, comfortable and user-friendly neonatal monitoring, wearable sensor systems should satisfy the following requirements [11]:

- Support fundamental health monitoring functions, such as monitoring and warning, and be safe to use in the NICU or at home.
- Be able to achieve continuous monitoring when the infant is inside an incubator or during Kangaroo mother care (Kangaroo care is a technique practiced on newborn, usually preterm, infants wherein the infant is held, skin-to-skin, with an adult).
- Be non-invasive and avoid disturbance of infants and avoid causes of stress or stimuli.
- Be scalable to include more monitoring functions such as wireless communication and local signal processing.
- Provide appropriate feedback that is interpretable for both parents and hospital staff on whether the system's components are functioning correctly.
- Look friendly, playful, and attractive to gain a feeling of trust from parents and clinicians.
- Be easy to dress and undress, and have easy-to-remove non-washable parts.

**Figure 1.**
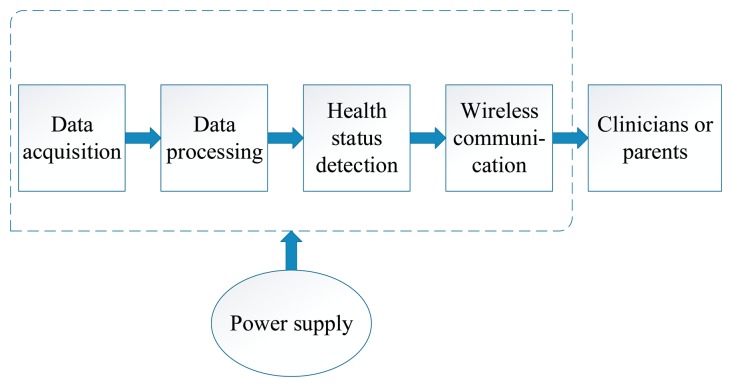
Framework of wearable sensor systems for infants.

An unobtrusive wearable sensor system for infants typically includes five modules: data acquisition or sensing module, data processing, health status detection, wireless communication and power supply [26,27]. The sensing module plays the essential role in acquiring data from infants by using different types of sensors or sensing elements. Data processing includes some data processing methods, such as A/D conversion, feature extraction, de-noising, amplification and some other algorithms. Health status detection can compare the results obtained by the sensor with the thresholds set by clinicians beforehand to determine the health status of infants. Wireless communication realizes the transmission of information between the infants, base station and the clinicians or parents. The power supply provides energy for the entire system. While wearable sensor systems have different approaches and methods to monitoring the health condition of infants, they basically share the framework described above, as depicted in Figure 1. The main five parts, including data acquisition, data processing, health detection, wireless communication and power supply, play a large role in wearable sensor systems, which will be introduced respectively in this part of the paper.

#### 2.2.1. Biomedical Sensor Modules

In the past, continuous monitoring of physiological parameters was only possible in a hospital setting. However, different types of sensors have been applied to obtain the physiological parameters of infants, which have the potential to be used in home environments. To achieve the goal of being effective and non-invasive, sensors used on infants have continued to be improved in recent years. Presently, most wearable sensors for infants pay attention to vital signs, including ECG, body temperature and respiration, which are the critical physiological parameters or vital signs in NICU care. The main limitations of sensors used in health monitoring for infants include the location of the sensor on the body and their fixation with garments to reduce the impact of motion artifacts, the integration with other sensors, interference form the external environment (such as temperature, humidity, sound, *etc.*), how to be small in size and low cost, how to be unobtrusive and have low power consumption [28], *etc.* People have to take these limitations into account during the design of a wearable sensor system. The main sensors in current use are explained briefly here, together with some sensing principles and techniques.

##### Electrocardiogram (ECG)

**Figure 2.**
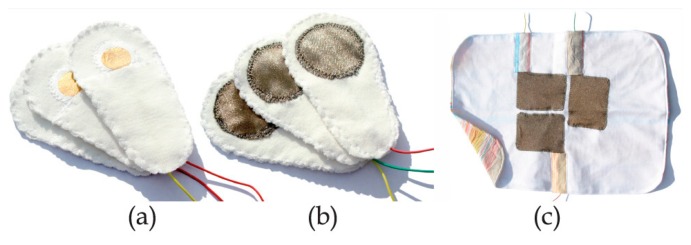
Textile electrodes for ECG measurement [30]. Copyright © 2010 Multi Science Publishing.

ECG, which is a widely-studied biosignal and describes the electrical activity of the heart, usually consists of QRS-complex, P-wave and T-wave [29]. The continuous monitoring of ECG is the most widely-used technique for cardiorespiratory monitoring in the NICU. Clinicians and researchers utilize gel electrodes attached to the skin to measure patients' biosignals. However, traditional methods of biopotential measurements may disturb or even harm infants. In the past, ECG was measured through gel electrodes attached on the body, but the long-term use of gel electrodes may cause skin irritation and allergic contact reactions and the electrical wires can also easily hinder both infants and the nursing staff. Because of the possible skin damage and wire disturbance to the vulnerable infants, we prefer to avoid the use of traditional gel electrodes in the ECG measurement of infants. The development of textile electrodes, including advances in conductive fabrics, metal-coated textiles and flexible electronics, makes biosignal measurements more effective and human-friendly. Some researchers use metal (such as silver)-coated polyurethane fabrics (one of the best textile materials for ECG measurement) to develop textile electrodes [19]. One kind of silver textile electrode integrated with a smart jacket has a knitted structure and can be mounted in different positions in the jacket to achieve good measurement results [30]. Figure 2 shows two patches with different versions of gold and silver textile electrodes (a and b), and a blanket with large silver electrodes (c). The textile electrodes usually have a knitted structure with the metal material knitted or woven through textile fabrics into cloth. Reliable integration into clothing may make the ECG measurement convenient and cozy for the infants by avoiding direct contact between the infants and the wires, electrical elements and circuits or utilizing textile electronics and conductive fabrics instead of traditional devices and hard wires. However, textile electrodes also have some disadvantages. For example, textile electrodes with a knitted or woven structure are usually bad quality and have poor skin-electrode contact, as well as their increasing sensitivity to motion artifacts [28]. In addition, the effect of abrasion and laundering on the fragility of the metal remains a problem in textile electrodes. Therefore, good methods for fixing textile electrodes to cloth and optimized ECG measuring methods are necessary.

**Figure 3.**
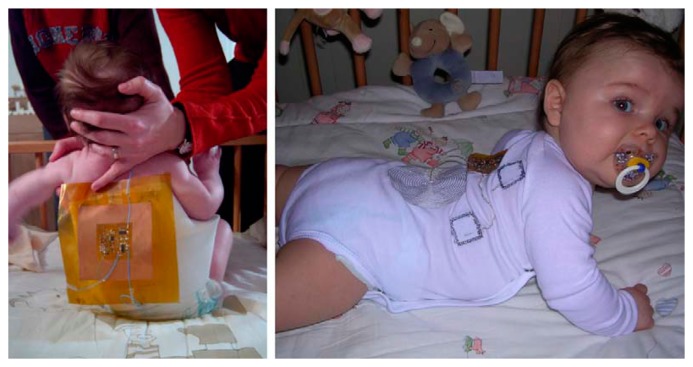
**Left**: demo set-up with belt prototype, worn by a 12-week-old baby; **Right**: baby suit prototype, worn by a 21-week-old baby [31]. Copyright © 2005 Elsevier B.V.

**Figure 4.**
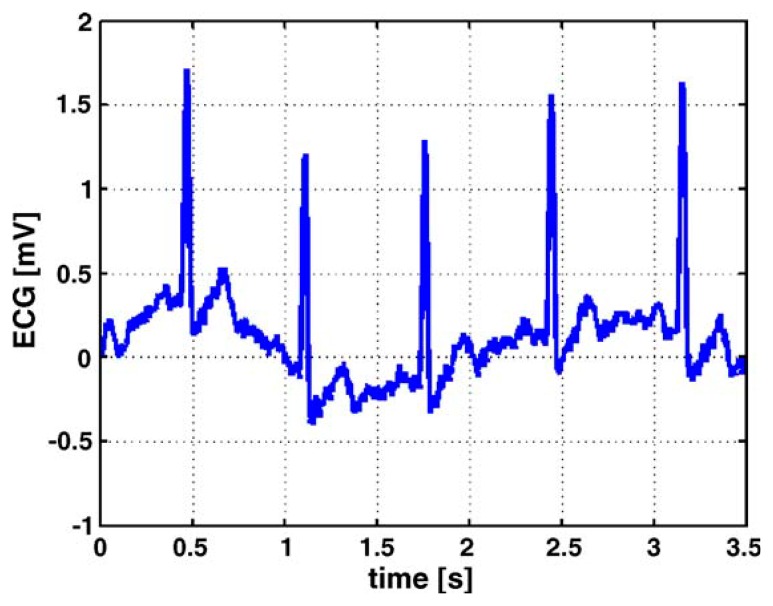
ECG measured with textile electrodes (in the same setup of the belt prototype in Figure 3) [31]. Copyright © 2005 Elsevier B.V.

Figure 3 is an example of ECG measurement, which shows two different kinds of test setup for measuring the ECG of infants. The belt prototype on the left utilizes textile electrodes knitted into an elastic belt. The belt wraps around the infant's body and can be worn under normal clothing; the elasticity of the belt assures good contact between the body and the electrodes, which effectively reduces the impact from the movements of the body. As an alternative to traditional ECG electrodes, textile electrodes, which are usually knitted or woven stainless steel electrodes integrated into the belt, are less irritating and have a lower toxicity than traditional gel electrodes. The yellow part of the belt on the infant's back is the flexprint with integrated circuitry and secondary coil (for power supply). The prototype on the right has all the circuit including the flexprint, coil, conductive wires and textile electrodes embroidered in the baby suit, and has a smaller size compared to the belt on the right, which further enhances the comfort of the infant. Figure 4 depicts a raw ECG measurement result using the belt prototype.

**Figure 5.**
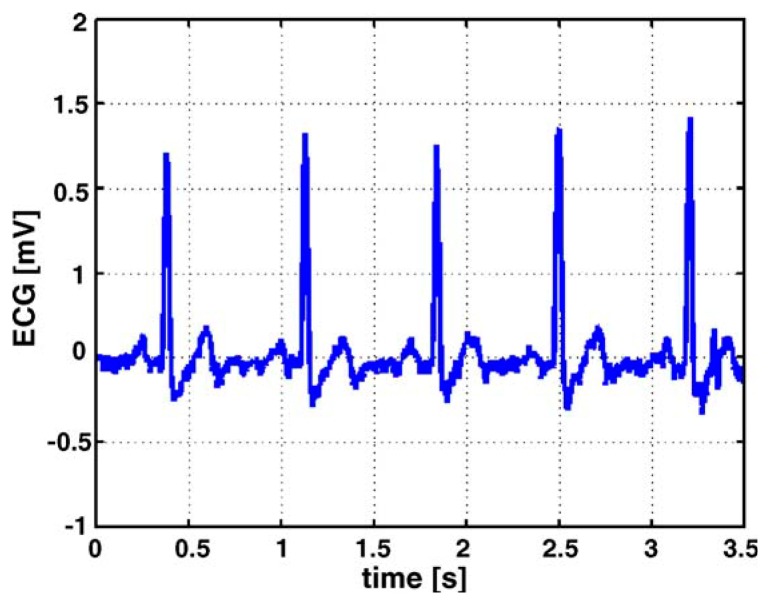
ECG measured with conventional electrodes (in the same setup of the belt prototype in Figure 3) [31]. Copyright © 2005 Elsevier B.V.

Figure 5 describes the ECG measurement in the same setup, except that it uses conventional electrodes instead of textile electrodes. From the comparison between the two test results, it is clear that the ECG signals measured with the textile electrodes display lower frequent base line drift, and the stability is lower than that of conventional gel electrodes. Nevertheless, textile electrodes can bring much comfort to the infants, and the signal quality is more than adequate for an accurate beat-to-beat detection of the heart rate, as demanded by the application [31].

##### Body Temperature Sensing Modules

**Figure 6.**
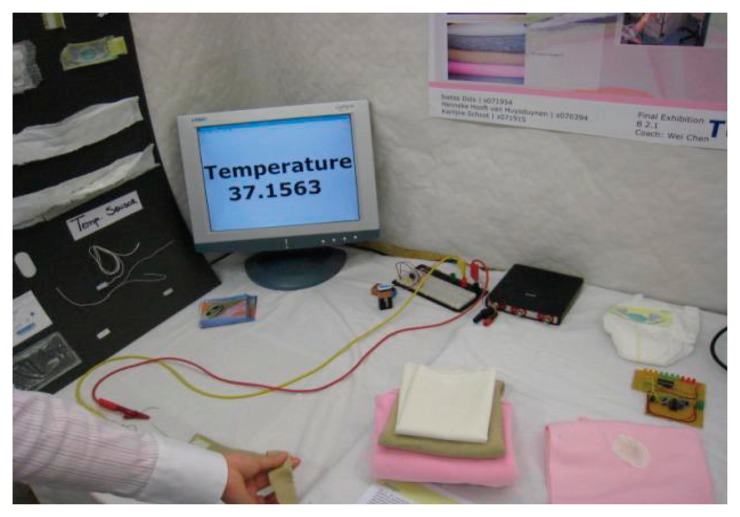
The setup of the temperature monitoring demonstration [10]. Copyright © 2010 Association for Computing Machinery, Inc.

**Figure 7.**
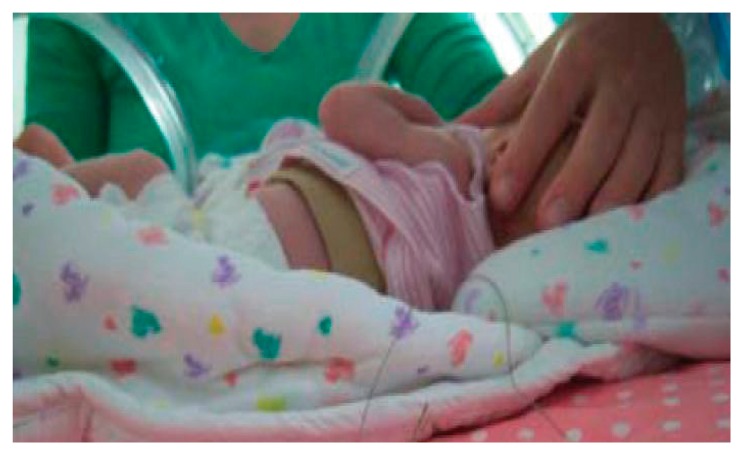
User testing on an infant in the NICU [10]. Copyright © 2010 Association for Computing Machinery, Inc.

Body temperature is one of the key physiological parameters in infant monitoring. Traditional methods of temperature monitoring are mainly through thermometer, which is very inconvenient and cannot achieve long-term temperature monitoring for infants. Constant monitoring of temperature can be achieved by using various kinds of electrical devices, such as thermistors or thermocouples, as the sensing elements. The accuracy of the thermistors and thermocouples is very high, which can achieve ±0.5 °C and ±0.1 °C, respectively. In the past, the placement of these temperature sensors and the presence of all the electrical wires easily lead to discomfort and even painful stimuli when the sticky sensors have to be removed. However, with the development of integrating technologies, these temperature-sensitive devices are integrated into a neonatal monitoring platform or smart clothing for temperature measurement. For example, a negative temperature coefficient (NTC) resistor applied as a temperature sensor has been introduced in Dols and Chen's research work [10]. The belt is able to wrap around the infant's body to achieve temperature measurement in a comfortable way. The setup of the temperature monitoring demonstration of the belt, as shown in Figure 6, consists of the prototype belt, data processing circuit, digital oscilloscope and a PC as a display for the monitored temperature. The researcher held the sensor on the belt and the temperature of his hand was displayed on the screen immediately, which was very convenient and effective. As depicted in Figure 7, the prototype belt was placed around an infant's body in the NICU to test to see whether the signals from the belt remained stable for a longer period during continuous temperature monitoring; the soft conductive wires connect the NTC sensor with the external circuit. With the belt wrapped around the infant's body, clinicians can easily know the temperature changes in the infant according to the measured results displayed on the screen. Continuous skin temperature measurement requires the sensing device to keep good track of the patient. Alternatively, infrared pyroelectric detectors can easily take a rapid one-shot temperature measurement from the ear. However, the accuracy of the latter is much worse, which remains a major concern [32]. To achieve long-term and accurate monitoring of a baby's core temperature, most temperature sensors are integrated into infants' underclothes. The measurement of axillary temperature, oral temperature and rectal temperature is limited because it is invasive and could easily cause anxiety in the infant. Apart from thermistors and thermocouples, temperature-sensitive fiber sensors, which contain temperature-sensitive fluorescent materials, and pyroelectric detectors, which convert the changes in incoming infrared light to electrical signals, are becoming popular in temperature monitoring. These sensors can achieve an accuracy of ±0.1 °C.

##### Respiration

As one of the basic approaches to evaluating the health status of patients, respiratory monitoring is very important for the infants in the NICU. Though respiration can be monitored in a number of ways, it can be generally classed as contact or noncontact.

For the contact method, sensing devices are attached to the patients' bodies. The parameters usually measured in the contact methods are respiratory sounds, respiratory airflow, respiratory-related chest or abdominal movements, respiratory CO_2_ emission and oximetry probe SpO_2_ [33]. For instance, Corbishley and Rodriguez designed a wearable respiration monitoring system which uses a miniature microphone mounted on the neck to obtain the breathing acoustic [34]. An electrical strain-gauge around the circumference of the chest can achieve respiratory monitoring on the basis of the resistance changes of the strain-gauge caused by the increase and decrease of the chest during respiration [9].

**Figure 8.**
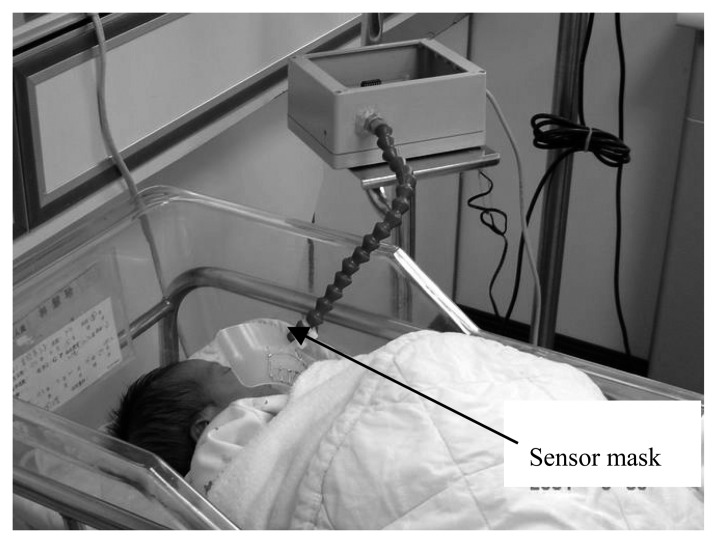
The adjustable sensor mask with thermo-sensors [36]. Copyright © 2015 World Scientific Publishing Co.

For the noncontact methods, some researchers made use of CO_2_ sensors placed around the crib on the bars to provide sufficient information about respiration [35]. There are many commercial off-the-shelf CO_2_sensors in the market with various sensing principles, such as electrochemical-based, infrared-based and metal-oxide-based sensors. The electrochemical based sensors give a better performance but have a short lifetime, and infrared based sensors are sensitive but bulky in size and more costly. The metal-oxide sensors are low cost but susceptible to the effects of temperature and humidity [13]. A thermal sensor-based respiration rate monitoring system was proposed in Hsu and Chi's work, which is another good example of a noncontact respiration monitor. Thermo sensors placed on the mask were sensitive to the temperature changes induced by respiration and the sensing data were collected and analyzed simultaneously by a personal computer, which can be linked to the central nursery room. As shown in Figure 8, by utilizing the thermo-sensors array, the sensor mask has the ability to perceive the temperature changes induced by respiration, and the ellipsoid-shaped mask was produced to avoid missing detection; it is also adjustable to accommodate different directions of the infant's face [36].

The main disadvantage of contact monitoring methods is that they require direct connection to the infant, some to the facial area or the belly, which disturbs many infants and may be poorly tolerated. Compared with the contact method of respiration monitoring, the non-attached monitoring method has the advantage of having no contact with the infant's skin, avoiding any possibility of skin irritation or other hazards. However, noncontact monitoring devices may be more complex and are susceptible to interference from the external environment (such as temperature changes, gas flow and breathing of the nursing staff or parents). Currently, noncontact respiration monitoring methods have not yet reached the level of maturity that can be used routinely in clinical environments. Concerns related to infants safety, electromagnetic interference with existing medical equipment, and complexity of operation have been factors in their slow take up in clinical environments [33]. With the development of technologies, these two different respiration monitoring will complement each other to achieve effective and reliable respiration monitoring. And the respiration sensors applied in the wearable sensor systems will become miniature, intelligent, user-friendly and accurate in the future, which makes the respiratory monitoring of infants credible and convenient.

#### 2.2.2. Data Processing and Health Status Detection

Data processing is considered as a way to collect and manipulate the sensing data to produce meaningful information. Generally, with enough memory for storing the sensing data, sensor networks are able to generate continuous, never-ending streams of data in health monitoring until they run out of power or break down. Data processing is an essential data mining method for acquiring useful information. The approaches of data processing mainly include calibration, denoising, sensor-level data fusion, A/D conversion and amplification. Data processing devices or circuits, such as transmitters, A/D converters, and filtering circuits, are widely used in wearable sensor systems.

Health status detection can compare the results obtained by the sensor with the threshold set by clinicians beforehand to determine the health status of infants. Generally, the stream of data from sensors is converted to a personal computer and the software installed on the PC processes the sensing data and judges the health condition of patients. The specialized algorithms applied in the software help to achieve optimal and reliable results, which will be sent to the clinicians or parents by wireless communication. Generally, the health status detection part contains an alarm system, which can send alarm information to clinicians or parents through wireless device (such as mobile phones and Bluetooth) whenever the infant meets an emergency. It is noteworthy that the false alarm may disturb clinicians and parents, therefore the optical algorithms and the level of sensitivity of sensors should be taken into count.

#### 2.2.3. Wireless Communication and Power Supply

In some wearable sensor systems in infant monitoring, the data interaction among sensors, computers and clinicians or parents is through electrical wires. These electrical wires can easily hamper the infant's movement, limit the distance between the infant and external circuits, and are usually difficult to sort out, which brings much trouble to infants and clinicians. The development of wireless sensing technologies such as Wi-Fi, wireless monitoring sensors, antenna and mobile phones with relative wireless protocols makes health monitoring more portable, convenient, economical and easier to install [37]. With the application of wireless communication, infants feel more comfortable during health monitoring and clinicians or parents are warned immediately through mobile phones when the health condition of an infant is at risk.

The wireless sensor network (WSN), based on wireless communication, sensor design and energy storage technology, has enabled the creation of a new generation of wearable sensor systems [38]. With the wireless interface between sensor nodes placed in different locations, the smaller, cheaper and more intelligent sensors are able to communicate with each other to form a network. WSN tries to provide an ideal wireless transceiver for the networking of human body sensors and ubiquitous health monitoring systems. Though these integrated microsensors in WSNs are only millimeters in size, they are capable of onboard processing and data interaction. With the rapid development and expansion of smart devices in recent years, WSNs have been widely applied in wearable sensor systems in our daily lives. WSNs have various monitoring applications, such as health monitoring, motion monitoring, power monitoring, habitat monitoring and so on. Based on the two sensor network mote technologies Tmote Sky and SHIMMER (Intel Digital Health Group's Sensing Health with Intelligence, Modularity, Mobility, and Experimental Re-usability [39]), Chris R. Baker *et al.*, developed five prototypes for home healthcare including SleepSafe, Baby Glove, FireLine, Heart@Home and LISTSENse [23].

To obtain reliable and continuous power supply for sensors, signal filters, amplifiers and other electrical circuits are a key consideration in wearable sensor systems. Not only do they need to meet the sufficient energy requirements and be easy to recharge during usage, the power also needs to be miniaturized, safe, and noninvasive. Wei and Sonntag proposed the innovative power supply “PowerBoy” for infant monitoring in the NICU, which is based on the principle of inductive Contactless Energy Transfer (CET). CET is the process in which electrical energy is transferred between two or more electrical devices through inductive coupling as opposed to an energy supply through conventional “plug and socket” connectors [27]. The “PowerBoy” used contactless power and a rechargeable battery embedded in a plush toy [40]. Additionally, some rechargeable batteries integrated into the clothes are also applied to the power supply of wearable sensor systems. Recently, researchers have been trying to replace traditional power supplies, such as batteries, with innovative power resources, such as human power and mechanical energy.

## 3. Methods or Techniques in Wearable Sensor Systems for Infants

In recent years, a great deal of attention has been paid to wearable sensor systems for the health monitoring of infants in the NICU or in the home environment. With the development of medical technology, sensor technology and wireless communication technology, the research in wearable sensor systems for infants has made a lot of progress. Wearable sensor systems are becoming smaller, more intelligent and many of them have been commercialized, which benefit numerous infants around the world. Various kinds of monitoring methods and techniques, such as direct monitoring, indirect monitoring, multi-parameter monitoring, single-parameter monitoring, textile technology, integration, wireless sensing and power supply, have been applied in these systems. In this section, methods or techniques in wearable sensor systems for infants are reviewed separately based on some typical systems which have been proposed by researchers.

### 3.1. Monitoring Methods

To obtain various kinds of parameter monitoring in the NICU or in the home environment, researchers have developed different kinds of methods for the healthcare of infants.

#### 3.1.1. The Direct Monitoring Method and Indirect Monitoring Method

**Figure 9.**
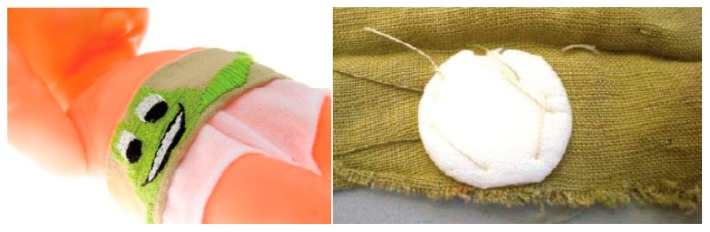
The prototype belt on a baby doll (**left**); and the isolation of the sensor in the belt (**right**) [10]. Copyright © 2010 Association for Computing Machinery, Inc.

**Figure 10.**
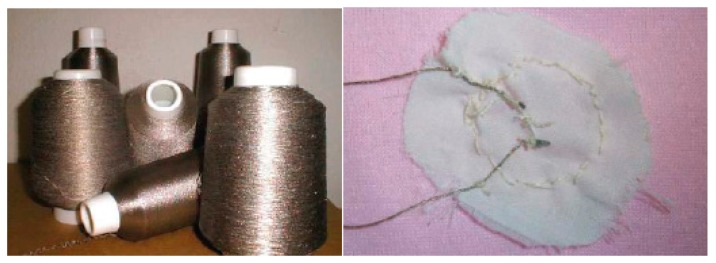
The conductive textile wires (**left**) and the connection of the sensor and the conductive wires (**right**) [10]. Copyright © 2010 Association for Computing Machinery, Inc.

The direct monitoring method is to monitor the physiological parameters or vital signs through sensors in a direct method in order to evaluate the health status of infants and detect life- or health-threatening events. Temperature and ECG are the regular parameters that can be easily and directly monitored by appropriate sensors. Chen *et al.* [10] proposed a prototype belt which was built of soft bamboo fabrics integrated with an NTC sensor to demonstrate direct temperature monitoring. The belt can be comfortably wrapped around the infant's body, and the NTC sensor is stitched to the belt and isolated using soft cotton to lessen the external temperature influence, as depicted in Figure 9. Since the NTC sensor is directly and tightly attached to the infant's stomach and is located above the liver to obtain optimal body temperature measurements, it is effective and convenient for acquiring the baby's core temperature of the body. Another novel and distinctive part of this belt is that flexible and soft conductive textile wires are used to connect the NTC sensor and external circuits instead of hard wires, which improves the comfort of the infant. Shieldex^®^ Silver Plated Nylon yarns woven as conductive textile wires and the connection of the NTC sensor and wires are shown in Figure 10 on the left and right, respectively. The accuracy of this belt can achieve 0.1 °C compared with the standard patient monitor according to experiments and user texts. As a direct and non-invasive way of the temperature monitoring of infants, the belt is convenient to use and can be integrated into a monitoring platform, such as the neonatal smart jacket. However, the connection between conductive wires and the temperature sensor is not reliable, which could be improved with more advanced binding or soldering techniques. In addition, the system can also be improved by utilizing wireless sensor network to replace the information exchange method through wires, which can enhance the comfort of infants.

**Figure 11.**
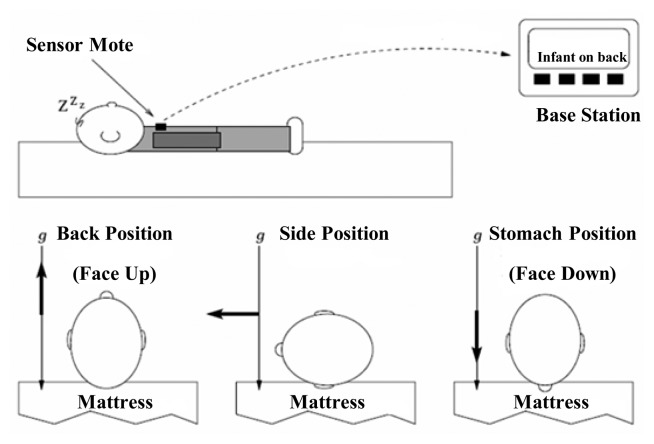
SleepSafe infant monitor [23]. Copyright © 2007 IEEE.

Alternatively, the indirect monitoring method is to monitor an infant's health condition through physiological activities and some special external environmental changes. For example, the sensors integrated into the clothing are able to determine sweating conditions according to the humidity of the patient's underclothes, and the detector installed on a walking stick can dynamically detect falling and measure pace of the elder people by utilizing a gyroscope chip to measure the angular velocity of the stick [26]. Chris *et al.* [23] developed a SleepSafe prototype utilizing a triaxial accelerometer attached to an infant's clothing to detect if the infant was sleeping on their back, side, or stomach. The prototype consists of an accelerometer sensor mote to monitor sleep condition and a wireless base station to receive and process sensor data, as depicted in Figure 11. The sensor mote is a SHIMMER mote and it is expected to be integrated into the fabric of the clothing with the technology trends of miniaturization. Sensor motes are created as an open-source hardware and software platform, combing sensors, low power wireless communication, and processing into a single architecture. These motes are also designed to have the ability to “self-organize” (resulting in a self-configuring WSN), and carry out onboard signal processing and distributed inference tasks prior to sending relevant information to a central controller [38]. The three different positions (back, side, and stomach) are measured as the opposite direction, perpendicular and aligned in the same direction to that of gravity, as described in Figure 11. With a small TinyOS program, the sensor mote's processor reads the triaxial accelerometer sensing data periodically via the on-board analog-to-digital converter (ADC), packetizes the data, and sends the packet wirelessly to the base station for processing.

**Figure 12.**
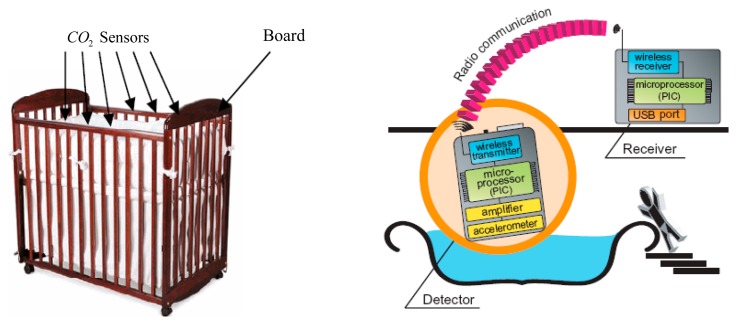
A crib with CO_2_ sensors mounted around the baby bed (**left**) [13] Copyright © 2007 IEEE; and a drowning prevention system based on a wireless accelerometer (**right**) [24]. Copyright © 2007 IEEE.

Some other non-wearable sensor systems for the indirect monitoring method have been proposed in recent years. Hung *et al.* [13] proposed a crib designed with metal-oxide based CO_2_ sensors implemented in the bars around the baby to monitor the infants' respiration, as depicted on the left of Figure 12. The processing board, which contains sensor signal processing circuits and comparators, is used to transmit and receive data. By monitoring the outputs of these CO_2_ sensors, researchers can detect whether there is anything unusual with the infant's respiration. Different kinds of tests have been carried out to verify the function of the crib with regard to various head directions and positions of the infant, and several disadvantages of this respiration monitoring system were pointed out. For instance, the outputs of a certain sensor and two different sensors were not repeatably identical for the environment with same concentration of gas. In addition, the output signals drifted over time. In addition, the functions of the metal-oxide CO_2_ sensors were affected by humidity and temperature. Therefore, it is necessary to find a sensitive yet stable sensor with performance uniformity and take some measures (such as feedback control) to reduce the impact from humidity and temperature on the sensors. As another example of indirect monitoring, a triaxial accelerometer was adopted to measure the water motion of a bathtub by detecting abnormal wave motion and giving a warning to guardians via a wireless communication receiver to prevent the infant drowning in the bathtub, as depicted on the right of Figure 12. According to the experiments conducted in real homes, the researchers acquired the data on wave motion during bathing, which can be used to distinguish a normal signal which occurs during a bath and an abnormal signal caused by drowning [24].

#### 3.1.2. Multi-Parameter Monitoring and Single-Parameter Monitoring

**Figure 13.**
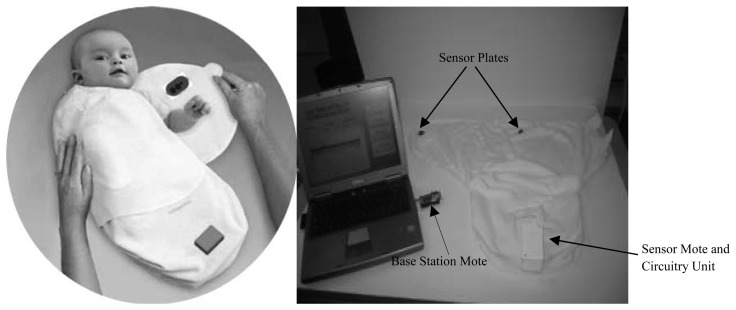
Baby glove swaddle (**left**) and the basic setup (**right**) [23]. Copyright © 2007 IEEE.

**Figure 14.**
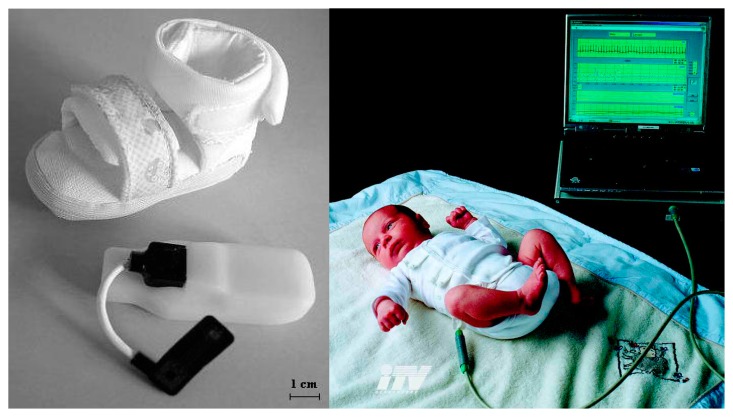
The BBA bootee (**left**) [9] Copyright © 2007 IEEE and the sensory baby vest (**right**) [12] Copyright © 2006 IEEE.

Multi-parameter monitoring provides a comprehensive, safe, reliable and accurate healthcare service, especially for the premature infants in the NICU. For instance, the baby glove swaddle [23] depicted in Figure 13 wraps and secures the child comfortably with two plates which contain sensors inside that monitor temperature, hydration and pulse rate – three main health considerations important to the development of an infant. The sensor mote, which contains a thermistor temperature sensor along with electrodes that monitor the infant's pulse rate and hydration, and the circuitry unit are integrated into the glove. The sensor plates are placed posterior and anterior to the child's upper torso, which is the body's largest thermal mass. The wireless base station allows the information exchange between the sensor mote and a variety of computing systems such as PDAs, cell phones and laptops, which allows parents or nurses to be alerted when the child's vital signs have exceeded predefined health settings. The BBA bootee [12] shown on the left of Figure 14 has been developed based on an oximetry module and a triaxial accelerometer to achieve multi-parameter monitoring for infants. Sensors data connection and transmission are managed by a microcontroller transceiver through a short-wave radiofrequency link. The adjustable straps made of elastic textiles ensure contact between the SpO_2_ sensor and the skin and permit the bootee to be fitted to the foot as the infant grows from one size to another. With the sensors integrated into the bootee, it is able to measure the pulse oximetry and detect the motion and the prone position of infants. In addition, with fully integrated sensors, the sensory baby vest [9] on the right of Figure 14 could also achieve multiple parameter monitoring in the NICU or in the home environment, and the parameters may include respiration, heart rate, temperature and humidity, e.g., by sweating. By utilizing different sensors integrated together, vital signs such as PPG, ECG, systolic and diastolic blood pressure, heart rate and body temperature can be monitored at the same time, and the data collected by the integrated sensor system can be correlated to form an overall framework of the infant's health, which greatly improves the efficiency of clinicians' work. However, multi-parameter monitoring also have problems with large energy consumption and mutual signal interference between different sensing modules, which need to be solved in the future.

**Figure 15.**
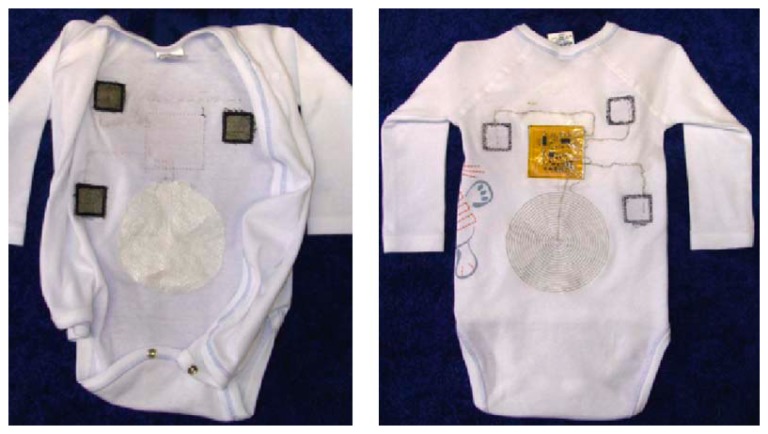
Baby suit prototype for ECG measurement. (**Left**) inside showing the Textrodes; (**right**) outside [31]. Copyright © 2005 Elsevier B.V.

Single parameter monitoring is monitoring health status through one pivotal parameter, which can simplify the healthcare monitoring system and increase the comfort of infants. The thermometer, sphygmomanometer and other medical measuring instruments mostly utilize the principle of the single parameter method. In order to alleviate the risk of the SIDS of infants (especially for preterm infants), a constant monitoring measurement of ECG is necessary for the premature infants in the NICU. Coosemans *et al.*, reported some prototypes intended for continuous monitoring of the ECG of infants using a garment embedded system [31]. All electronics including textrodes (textile electrodes) and inductive coils are mounted on a flexible circuit to facilitate integration in the infant's pajamas, as depicted in Figure 15. The circuit and conductive wires are embroidered in the infant's suit for the purpose of comfort. This system only has three pieces of textile electrodes for single ECG measurement, which makes the data processing circuit simple and physically small. The features of this kind of single parameter monitoring device usually contain only one kind of sensor to measure the specific parameter, and the circuit is simple, which leads to low power consumption. The single physiological parameter monitoring method shows great potential in reducing the risk of SIDS, usually caused by cardiac arrest, which can be effectively monitored through ECG monitoring.

### 3.2. Related New Technologies for Wearable Sensor Systems for Infants

With the development of various new technologies applied in wearable sensor systems for infants, such as intelligent devices, wireless sensing systems and integration technologies, the healthcare systems for infants are becoming more reliable, comfortable, intelligent and convenient. In this part of this paper, some essential and novel technologies in wearable sensor systems are reviewed.

#### 3.2.1. Textile Technology

**Figure 16.**
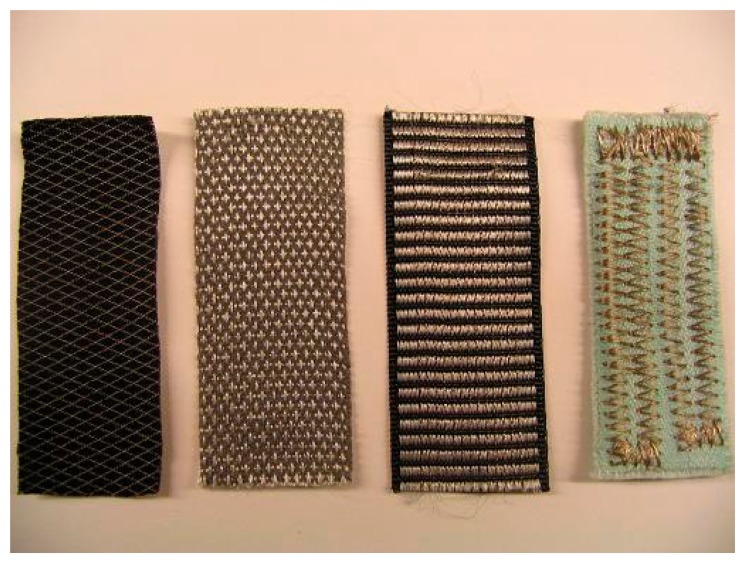
Four textile electrodes with different structures and materials [29]. Copyright © 2007 IEEE.

Owing to the disturbance and discomfort caused by hard electrical wires or electrodes, many researchers have utilized textile wires or textile electrodes to replace conventional wires and electrodes, which have achieved good responses. The innovation in terms of textiles is related to the use of conductive yarn integrated into a fabric structure for sensing and acquisition of signals such as electrocardiogram, electromyogram, respiratory activity, skin conductivity, and index of motion. As shown in Figure 10, the conductive yarns can connect the sensors or electrodes with external circuits instead of hard wires through two main fabric manufacturing techniques—woven textiles and knitted textiles—which makes the monitoring system light-weight and comfortable. Textile electrodes are a new, attractive choice for biopotential measurements. These special electrodes are made from fabric with electrode appearance. Figure 16 shows four textile electrodes of different materials and structures. Generally, textile materials are insulators, but in textile electrodes, conductive yarn is weaved, knitted or embroidered into a fabric during the manufacturing process. Conductive yarn can be made, for instance, from silver-coated yarn or metal filaments can be braided into yarn [29]. With the seamless knitting technology, the elastic and adherent textile electrodes can be in close contact with the infant's skin [41]. However, the movements of the infant can lead to the poor quality of the data on physiological parameters while the textile electrodes are monitoring. To improve the electrical signal quality in dynamic conditions, a hydrogel membrane was used in Paradiso's work [42]. In addition, the high impedance in the electrode-skin interconnection also remains a major drawback of textile electrodes.

#### 3.2.2. Integrated Measurement Platform

**Figure 17.**
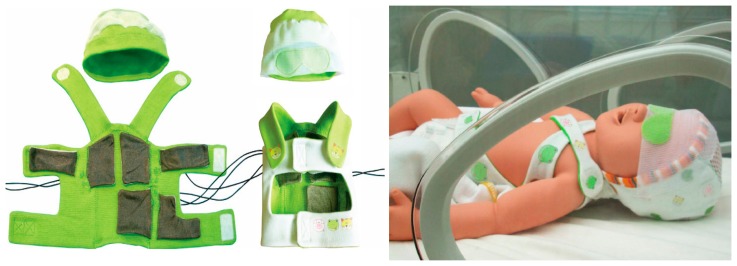
A prototype of the smart jacket (**left**) and a baby mannequin wearing the smart jacket inside the incubator (**right**) [11]. Copyright © 2009 IEEE

With the development of wireless communication technologies, advances in sensor technologies and intelligent devices in wearable electronics have enabled the creation of a new generation of healthcare monitoring systems for infants [38,43,44]. The integrated measurement platform allows different types of sensors or wearable electronics to be embedded, which makes the monitoring system's size smaller, more comfortable and more convenient to maintain. For example, the smart jacket [11] is an integrated platform for vital sign monitoring of newborn infants. Attached with flexible, lightweight textile sensors and electronics, it is convenient for the monitoring of ECG and respiration parameters for the jacket. Figure 17 shows the silver-coated textile electrodes integrated into the smart jacket prototype. The jacket is open chest and has an open structure fabric on the back for clinical observation. The jacket also consists of a hat for eye protection. The six textile electrodes, distributed in different positions of the jacket, can effectively reduce the poor signal caused by movement artifacts. The prototype design also allows freedom of movement, protection of eyes and gives an aesthetic effect, which is appreciated by parents and clinicians. In the future design, some other sensors, such as temperature sensors, sweat pH sensors and body odor sensors, can also be integrated into the jacket. Furthermore, it is meaningful that wireless data transmission is used to replace electrical wires in the consideration of comfort. In addition to the use of health monitoring, another integrated sensor system is proposed to support medical staff during cardiopulmonary resuscitation (CPR) of newborn infants. In this system, a prototype rhythm of life aid (ROLA) device was built, consisting of a transparent foil integrated with pressure sensor and electroluminescent foil actuators for indication of the exerted chest compression pressure, as well as an audio box to generate distinctive sounds as audio guidance for insufflations and compressions [45]. The clinicians can make appropriate adjustments during CPR according to the feedback from the audio signal buzzer and the visual actuator.

Though the integrated measurement platform has a great potential in wearable sensor systems for infants, there are still some limitations which require resolving. For instance, the continuous and reliable power supply remains a big problem caused by the high power consumption of various sensors and complex data processing circuits. And the hampering wires discomfort infants during their daily activities. In addition, the integrated platform has difficulty in keeping noninvasive and small size while having various monitoring units inside. In the future, the integrated platform needs to be improved to be rugged and unobtrusive, provide consistent and reliable data stream and have higher level medical algorithms implemented to classify the measurements results (such as normal, abnormal, risk, high-risk and error) and enable to transmit data through wireless sensor networks [46].

#### 3.2.3. Wireless Sensing Technologies

In recent years, wireless technologies including Bluetooth, Zigbee, GPRS, wireless Local Area Network (LAN) and Radio Frequency (RF) have already played a large role in wireless healthcare systems. For long-term healthcare systems, it is more convenient for the patients to have a wireless transceiver to transmit acquired and processed sensing data to an off-body host computer and control the functioning of the sensor system. Chen *et al.* simply described the conceptual system of infant health monitoring with a wireless device [47]. In wireless wearable sensing systems, signals from sensors are translated into a certain digital value by the microcontroller, and the wireless module wirelessly transmits the data in a continuous stream to a central processing unit/monitor to ensure real-time health monitoring by medical personnel. Wireless technology such as Bluetooth and Zigbee generally utilizes an appropriate wireless protocol and well-designed software for data communication with sensors and processing [48]. A wearable e-nose prototype was developed with the use of Zigbee wireless technology in a compact armband for monitoring the axillary odor released from the human body [49]. Currently, with the development of Wi-Fi technology and mobile phones, wireless technology has shown great potential to enhance healthcare monitoring performance in daily life. In order to achieve long-term monitoring and compact systems for infants, the key considerations for choosing wireless systems are the size and power consumption of the module.

WSNs have important applications in the health monitoring of infants. Sensor nodes in WSNs are capable of sensing, measuring, processing and transferring physical parameter data to the parents or clinicians conveniently and effectively. However, because of the limited power, processing capacity and storage of each sensor node, new communication protocols and management services are needed to fulfill these requirements. Hence, the development of a reliable and energy-efficient protocol is very essential and important for supporting various WSN healthcare applications. Yick *et al.*, reviewed the various energy-efficient protocols for the transport layer, network layer, data-link layer and the cross-layer connections in WSNs [39].

Generally, the adequate transmission range for clinic observations is 2–5 m, and for home care is 20–100 m. The range of Bluetooth is usually set to 10 m (with 0 dBm output power), and the Bluetooth chipset can be augmented with an external power amplifier to extend the range (up to 100 m with +20 dBm output power) to support home environment. The range of Wi-Fi and Zigbee can achieve 20–100 m, which is adequate for the basic application of health monitoring. In future, the wireless transmission range of sensing data to remote monitoring station needs to be improved with a low consumption of energy. Optimal communication protocol needs to be developed for the remote monitoring systems to achieve high information security and noise immunity. In addition, since the current quality and reliability of health monitoring with infrastructure-oriented wireless networks have not been very satisfactory due to the unpredictable and spotty coverage, more attempts should be taken with the infrastructure oriented wireless networks. *Ad hoc* wireless networks can be formed among mobile and wearable infant-monitoring devices for improving coverage of infants monitoring when infrastructure-oriented networks are not accessible with very high reliability [46]. The energy consumption of wireless transmission needs to be reduced and the reliable power supply for continuous data transmission needs to be developed.

#### 3.2.4. Power Supply

**Figure 18.**
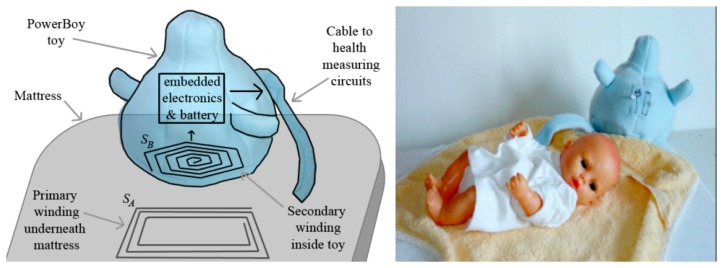
The Powerboy prototype (**left**) and the Powerboy near a baby mannequin (**right**) [40]. Copyright © 2009 IOS Press.

Reliable and continuous power supply for the sensors, micro-computers, signal amplifiers and transmitters used in wearable sensor systems is essential for the healthcare of infants. The power supply for health care systems should be non-invasive, inexpensive, reliable and rechargeable. There is already a variety of power supply systems in the marketplace, however almost all of these platforms were designed to run on batteries that have a very limited lifetime. The potential power sources for wireless sensor nodes may include batteries, micro-fuel cells, micro-heat engines, electromagnetic (RF) power, photovoltaics (solar cells), human power, wind/air flow, and thermal and mechanical energy sources [50,51]. The short-lived sustainability and high cost of power supplies is a significant limitation for wearable sensor systems in application. The power supply needs to continue to work for more than 1 day to be convenient. However, currently most batteries of the wearable sensor systems for continuous health monitoring on the market can only last nearly 2 h, or even less, which hardly meets the needs of users. In addition, the batteries which can last more than 4 h are usually very expansive. In order to minimize the maintenance and replacement costs of batteries, researchers have tried to develop innovative power solutions. Innovative materials, energy conversion and energy-scavenging technology (such as energy harvested from human sound, body heat and movement) have been taken into account in recent years to supply reliable power for wearable sensor systems. For example, Chen *et al.* proposed a wireless power supply system prototype for infants inside an incubator or in their parents' hug during Kangaroo mother care based on the principle of inductive contactless energy transfer [40]. The power supply design focuses on the contactless energy transfer system as well as the primary and secondary windings that generate the magnetic fields. As described on the left of Figure 18, when the PowerBoy plush toy is placed on the mattress above the primary winding, power is then transferred from the primary winding to the secondary winding through their mutual inductance. At the same time, the rectifier circuit and power converter charges a battery inside the toy, and supplies the monitoring equipment with power via a power cable inside the toy's fluffy tail. When the PowerBoy is lifted with the infant and the inductive link between the windings is broken, the circuitry inside the toy detects this and switches on the battery to power the monitoring equipment. As shown on the right of Figure 18, the Powerboy has aesthetic features and keeps some distance with the baby mannequin for non-invasive purposes, so this inductive solution for power supply has nearly no effects on the biomedical parameter (such as temperature) monitoring of infants.

It is noteworthy that apart from the electrical methods such as batteries, inductive methods and piezoelectric material, which is extremely popular in wearable power supply systems, the mechanical way of generating energy may be an innovative and effective way to achieve inexpensive and continuous power supply for systems. Just like mechanical watches, wearable sensor systems can continue to work a long time after the clockwork spring has been wound up. The small-sized, non-invasive mechanical design and the effective conversion of mechanical energy to electrical energy are the main limitations which need to be considered in the future.

## 4. Applications of Wearable Sensor Systems for Infants

With the development of sensor technology, wireless communication techniques and integration process, wearable sensor systems for infants have become a widespread and useful tool for both clinical practice and biomedical research. Using integrated platforms, low-cost wearable sensors and reliable power supplies, wearable sensor systems can be conveniently used in healthcare and physical development, as summarized in the remainder of this section.

### 4.1. Clinical Diagnosis and Healthcare Monitoring

Since infants cannot communicate with others by speaking, in the clinical diagnosis of cardiovascular diseases, cystic fibrosis or other diseases that are difficult to diagnose the clinicians need to acquire enough physiological information on infants. As a result, they have to consult corresponding measurement devices for help in making the correct diagnosis based on the estimation results of the device. With specialized sensors and integrated sensing systems, wearable sensor systems can easily monitor the vital signs or physical parameters of infants. Currently, reliable systems based on various kinds of novel sensors, such as piezoelectric transducer sensors [52], non-contact capacitive sensors [53], which are utilized to measure the biopotential of patients in order to monitor the ECG parameter, are used to achieve cardiorespiratory monitoring. For instance, the electro-optic (EO) acquisition system is designed for wearable ECG monitoring, and clear ECGs can be obtained with the EO sensor, which shows adequate and reliable clinical performance [54]. Additionally, thermistors or thermocouples are used in wearable sensor systems to monitor the core temperature of infants, which is a widely-used application of wearable healthcare systems. Pulse rate, respiration rhythm, and body movement in bed can also be monitored through relative sensors [55].

In addition, another application of wearable sensor systems with regard to healthcare is to prevent apparent life-threatening events in daily life. It is well known that fall injuries remain a major risk factor for infants at home. Triaxial accelerometers, gyroscopes, inertial sensors and other wearable devices help researchers to understand the underlying mechanisms and kinematics of falls. Three basic kinds of falls—falling from sleeping, falling from sitting and falling from standing—are identified and specified the characteristics of acceleration changes during fall progress [26]. According the features of different types of fall, approaches using sensors to detect fall injuries have been put forward; combined with wireless communication technology, such as mobile phones, parents are warned as soon as the fall injury happens. Krenzel *et al.*, proposed a wireless slip and fall prediction system after accurately analyzing and applying largest Lyapunov exponents (LLEs) calculated from accelerometer and gyroscope time series data acquired from falling and flipping to predict falls [56]. Apart from falling, home risks of infants, such as drowning, child abuse, fire danger and toxic gases, can also be monitored and avoided through wearable sensor systems applied on infants.

### 4.2. Ecological Behavioral Analysis and Physical Development

Ecological behavior of infant is the spontaneous infant's movement which is strictly linked to the brain development. Generally, spontaneous movements of newborn infants matter for developing the capability of generating voluntary skill movements. Thus, the ecological behavioral analysis of infants is very attractive to parents and researchers. Additionally, since infants cannot express their thoughts and feelings through words clearly, wearable sensor systems may help parents to understand what infants want through measurements of their body's vital signs or analysis of their ecological behavior, such as crying (crying has different types for infants in different situations), hand flapping and eye movement. Taffoni *et al.*, designed and developed a mechatronic wearable device for ecological movement analysis called Wrist and Ankle Movement Sensor (WAMS) [57]. The WAMS device was developed by using a micro-fabricated device integrating all the required sensing capability to be a kinematic sensing unit, including a triaxial magnetometer, accelerometer and gyroscope analog sensor. Another system for behavioral analysis named gaze-tracker is proposed to monitor gaze and head movements in infants. The gaze-tracker is a component of the Audio-Visuo-Vestibular-Cap (AVV-Cap), a multimodal headworn device designed for assessing social orienting behavior in children between 12 and 36 months. The sensors in the AVV-Cap include a magneto-inertial sensor and a mini-webcam (referred to as Eyecam) mounted on the visor of the cap pointing towards the face of the child. When the infant's hand is turning, the magneto-inertial sensor and the webcam are able to determine the position of the eye's gaze [58]. After measuring, recording and processing the data of the infants' movements or behaviors, the computer has the ability to judge the purpose of the child's behavior and tell parents or clinicians through wireless communication.

Moreover, wearable sensor systems can assist infants with physical development by monitoring their physiological parameters. The system is able to tell the caregivers which nutrition the infant is short of according to the monitoring results of the infant's physiological parameters. Wearable sensor systems can also be used to deepen links between parents and infants by monitoring specific parameters of infants to know their requirements and remind the parents to give them some feedback at the appropriate time, which is a very meaningful application for the development of infants.

## 5. Conclusions

As a significant tool in clinical practice and human behavior research, wearable sensor systems have attracted an increasing amount attention from researchers and clinicians since the 1970s. Associated with the development of biomedical, wireless communication and computer science, wearable sensor systems based on sensing technology have created a new generation of constant health monitoring and have been widely used in healthcare and behavioral analysis for infants in recent years. In various ways, such as successful integration into infant's clothing and textile electronic devices, wearable sensor systems are becoming unobtrusive and noninvasive to infants. In addition, wearable sensor systems utilize wireless communication systems to link infants and parents or nurses, which is very convenient and reliable in the healthcare of infants. At present, commercialized wearable sensors have been adopted in various applications of wearable sensor systems for infants.

The current paper provides a systemic and detailed review of wearable sensor systems for infants. After the introduction of the significance and role of the wearable sensor systems in continuous health monitoring for infants, the main basic monitored parameters in clinical practice and daily life and the framework and main modules utilized in these systems, which constitute the basis of wearable sensor systems for infants, were summarized. Monitoring methods and techniques, such as single-parameter monitoring, multi-parameter monitoring and textile electrode technology in wearable sensor systems, were reviewed according to some recent research and applications in the field. Significant application prospects for the healthcare and physical development of infants can be expected and exploited.

Until now, continuous healthcare and behavior analysis for infants using wearable sensor systems has made great progress and shown good application prospects. In order to realize the wide application of this technology in our life, some detailed technical matters still need to be improved, such as the reliability of the sensing results of the wearable sensors, which may be affected by many external influences, the optimal choice of analytical algorithms for stream data in different applications, reliable and long-lasting power supplies, the development of miniature sensing modules and miniature integrated platforms and wider wireless communication coverage, and so on. Intelligent wearable sensor systems in the future need to become low-cost and low-power and be able to integrate multiple wearable sensors and wireless data transfer within small volumes. Additionally, the research and creation of appropriate power supply sources is extremely important to continuous health monitoring system. Another important trend and research direction is to achieve communication and information feedback between parents or clinicians and infants through wearable sensor systems by making the wearable sensor systems become the platform for understanding the real-time needs of infants for caregivers. In addition, it is noteworthy that auxiliary equipment coordinated with wearable sensor systems for infants has the great potential to reduce the burden on parents when they take care of infants. Wearable sensor systems exchange the information acquired from infants with wearable auxiliary equipment, and the auxiliary equipment, such as actuators or protection devices, can start to work to help infants or parents to complete some actions, including wearing clothes, taking a bath or breastfeeding when necessary, which may greatly reduce the burden on parents or caregivers. Once these developments are achieved, not only can the infants receive continuous healthcare at a very low cost, but also the parents and clinicians can grasp the infants' requirements easily and conveniently and the work of child-care will become easy and effective, which surely will attract parents and clinicians and will be widely adopted in daily life, various clinical situations, and other possible applications of infant monitoring.

## Figures and Tables

**Table 1.** The main physiological vital signs and parameters monitored during neonatal intensive care, with relative sensing principles and transducers.

**Parameter or Vital Sign**	**Sensing Principles**	**Transducers**
ECG, EEG	Electrical, bio-potential	Skin metal electrodes, textile electrodes [19]

Heart rate, Pulse	Optical, pressure	Photodetector, force sensitive resistor [20]

Invasive Blood Pressure	Auscultatory	Pneumatic cuff & microphone

Temperature	Electrical, resistance	Thermistor
	Electrical, thermoelectric	Thermocouple
	Optical, IR emission	IR pyroelectric detector
	Optical, fluorescence	Photodetector

Respiration	Mechanical, expansion	Strain gauge
	Electrical, impedance	Skin electrodes

SpO_2_	Optical, absorption	Photodetector & emitters (red and IR)

PO_2_	Optical, fluorescent	Photomultiplier tube
	Electrochemical, amperometric	Clark oxygen electrode

PCO_2_	Optical, fluorescent	Photomultiplier tube
	Electrochemical, potentiometric	Ion-sensitive electrode

Glucose	Optical, colorimetric	Photodetector
	Electrochemical, amperometric	Enzyme modified biosensor

Lactate	Optical, colorimetric	Photodetector
	Electrochemical, amperometric	Enzyme modified biosensor

Bilirubin	Optical, colorimetric	Photodetector

Urea	Optical, colorimetric	Photodetector
Electrolytes:-	Optical, colorimetric	Photodetector

Na^+^, K^+^, Ca^2+^, Cl^−^	Electrochemical, potentiometric	Ion-selective electrode (ISE)

pH	Optical, colorimetric	Photodetector
	Electrochemical, potentiometric	Ion-sensitive electrode

Haematocrit	Optical, reflectometric	Photodetector
	Electrical, impedance	Aperture-tube electrode

Haemoglobin	Optical, absorption	Photodetector and emitters
